# Validation of Assessment of Medication Swallowability by Surface Electromyography Measurement: Evaluating the Effect of Capsule Size on Medication Adherence

**DOI:** 10.7759/cureus.96442

**Published:** 2025-11-09

**Authors:** Tomohisa Kato, Mitsuki Yamamoto, Yoshinori Sasaki, Shouji Yokota, Takashi Eto, Akihiro Onishi, Naomi Kurata

**Affiliations:** 1 Clinical Development, Sawai Pharmaceutical Co. Ltd., Osaka, JPN; 2 Research and Development, Sawai Pharmaceutical Co. Ltd., Osaka, JPN; 3 Clinical Pharmacology, Medical Co. LTA, Hakata Clinic, Fukuoka, JPN; 4 Laboratory Medicine, The Jikei University School of Medicine, Tokyo, JPN; 5 Pharmacology, School of Pharmacy, Showa Medical University, Tokyo, JPN

**Keywords:** capsule, ease of swallowing, electromyography, medication adherence, questionnaire evaluation

## Abstract

A decrease in adherence leads to a reduced treatment effect, and improving adherence in treatment is a significant issue worldwide. Medication non-adherence is caused by a variety of factors, and difficulty in swallowing medications has also been suggested to contribute to non-adherence. Therefore, when developing a medicine, it is important to strive to make it easy to swallow, and it is equally important to correctly evaluate the ease of swallowing a medicine.

Subjective questionnaire evaluations, which are important evaluation systems, are mainly used to evaluate the ease of swallowing medicines. However, it is widely known that various biases (common method biases (CMB)) occur in questionnaire-based assessment methods, and it is difficult to eliminate them completely. In this study, we investigated a highly objective method for evaluating the ease of swallowing medicines using an instrument. Surface electromyography measurement of swallowing-related muscles, which is one of the methods for evaluating swallowing ability and the feeling of swallowing using an instrument, is a minimally invasive and simple method. In this study, we evaluated the ease of swallowing capsules in healthy adults and assessed whether the electromyographic activity of swallowing-related muscles can be used to evaluate the ease of swallowing a medicine.

The results showed a correlation between the amount of muscle activity (area value) when swallowing a capsule and capsule size. In addition, it was shown that there was a positive correlation between the questionnaire results and muscle activity. These results indicate that assessing the surface muscle activity of swallowing-related muscles is a useful method for evaluating medication adherence.

## Introduction

Improving medication adherence in treatment is a very important task. Some reports have shown that reduced adherence leads to lower treatment efficacy [1, 2]. WHO said in a report on adherence in 2003, "Increasing the effectiveness of adherence interventions may have a far greater impact on the health of the population than any improvement in specific medical treatments" [3]. It is necessary to analyze the factors of non-adherence and to try to find a solution for each factor. Medication non-adherence is caused by a variety of reasons [4] and has been reported to be influenced by the ease of taking medication [5, 6]. Therefore, the development of easy-to-take drugs is crucial for achieving treatment success through improved medication adherence.

In the development of an easy-to-swallow medicine, it is important not only to devise ways to make it easier to swallow but also to correctly evaluate the ease of swallowing the medicine. While questionnaire assessment is a key modality [7-9], it is common for questionnaire assessment to be associated with a variety of biases (common method biases (CMB)), e.g., impacts on respondents due to social desirability, etc., and effects of selected items, such as wording ambiguity. In addition, it has also been reported that it is difficult to completely eliminate CMB in questionnaire assessment [10]. Ease of swallowing medicine is an important factor in future drug development, so establishing new methods for evaluating ease of swallowing medicine that are superior to general questionnaires is one of the important challenges in the development of this field.

In this study, we examine a method for evaluating the ease of swallowing medications using a device as a more objective method than a questionnaire assessment. Surface electromyographic measurement of swallowing-related muscles has been reported as a method of assessing swallowing ability and the feeling of ingestion using instruments. It is also a simple method with low invasiveness. Surface electromyographic measurements of swallowing-related muscles have been utilized as a method to assess the ease of swallowing, as reported in the evaluation of differences in eating bolus [11] and the evaluation of differences in ease of swallowing depending on body position [12]. However, no results have been reported on whether it can be used as an evaluation system for the ease of swallowing medicines.

There are various factors affecting the ease of ingestion of drugs [13, 14]. In this study, we focused on the size of medicines, which is an important factor in ease of swallowing [15, 16], and used capsules in which the size was the only variable. Therefore, we conducted a clinical study to evaluate the ease of swallowing depending on capsule size in healthy adults and assessed whether the amount of muscle activity of swallowing-related muscles is useful as a parameter for evaluating ease of swallowing.

Furthermore, in research into drug swallowability, assessing swallowability by having subjects swallow drugs poses a high hurdle in terms of ethics and safety. Therefore, in this study, we decided to confirm how accurately swallowability can be assessed without actual ingestion. To do this, we had subjects evaluate their impression of apparent ease of swallowing prior to actually swallowing the drug. We then evaluated the correlation between their impression of apparent ease of swallowing and the actual ease of swallowing.

## Materials and methods

Sample

In this study, six capsule formulations of different sizes containing no pharmacologically active substances were used and manufactured under Good Manufacturing Practice (GMP) control. The capsules (Qualicaps Co., Ltd., Nara, Japan) were white in color, shells were made from hypromellose, the contents were lactose hydrate, and capsule formulations No. 0 to No. 5 specified in the Japanese Pharmacopoeia were used (Table 1).

**Table 1:** Capsule number and size

Capsule no.	#0	#1	#2	#3	#4	#5
Overall length (combined) (mm)	21.8	19.4	18.0	15.8	14.5	11.4
Inner diameter (mm)	7.33	6.63	6.08	5.56	5.06	4.66
Volume (mm^3^)	726.1	558.6	438.3	271.6	242.2	151.1

Subjects

This study was conducted in accordance with the "Declaration of Helsinki" and the "Guidance on Ethical Guidelines for Life Science and Medical Research Involving Human Subjects in Japan." The protocol and informed consent forms for this study were approved by the Hakata Clinic Clinical Studies Review Board in Fukuoka, Japan (reference number: 2133CP), prior to beginning the associated study procedures. This study was conducted at Souseikai Hakata Clinic with research funding from Sawai Pharmaceutical Co., Ltd., to which the authors are affiliated.

A total of 40 healthy Japanese male and female subjects aged 20 to 39 years were enrolled in the study (Table 2). The subjects were those who did not take drugs or supplements on a daily basis and were able to take No. 0 capsules. Before enrolling in the study, all participants provided written informed consent for participation in the study. They had been informed verbally and in writing about the purpose of this study, methods, and potential risks by qualified staff and had been allowed sufficient time to review the information provided and to receive answers to any questions they asked. All subjects were judged to be eligible for the study when assessed against the inclusion and exclusion criteria.

**Table 2:** Subject background (upper row: average, lower row: minimum-maximum)

	Sex	N	Age	Height (cm)	Weight (kg)	BMI
20's	Male	10	22.1	172.0	62.8	21.2
			20―27	166.3―185.5	58.5―73.1	19.2―22.2
30's		10	35.3	170.9	66.7	22.8
			30―39	164.6―181.7	55.1―79.8	20.3―24.6
20's	Female	10	23.5	159.4	54.5	21.4
			21―27	151.4―167.1	50.0―61.8	18.5―24.5
30's		10	33.7	159.1	54.7	21.5
			30―38	151.2―165.5	46.0―64.7	18.9―24.3

Clinical study

First, the subjects were asked to fill out a questionnaire (pre-questionnaire) to evaluate the apparent ease of swallowing each capsule based on visual information. After completing the pre-questionnaire, the subjects took the capsules, had their electromyograms measured, and completed another questionnaire (post-questionnaire).

The order in which the capsules were taken was random, and one capsule was taken with a 20 mL water-based solution. The interval between doses of capsules was five minutes or more, and after the impact of the capsule taken immediately before was eliminated, the next capsule was taken.

For both the pre-questionnaire stage and capsule-taking stage, subjects were randomly given one capsule numbered 0 to 5. When taking the capsules, the subjects were blindfolded, and the capsules were administered into the oral cavity by the staff. The interval between capsule intake was at least five minutes, and the next capsule was taken after any effects, such as the feeling of the previous capsule sticking in the throat, had subsided.

Electromyographic measurement

**Figure 1:**
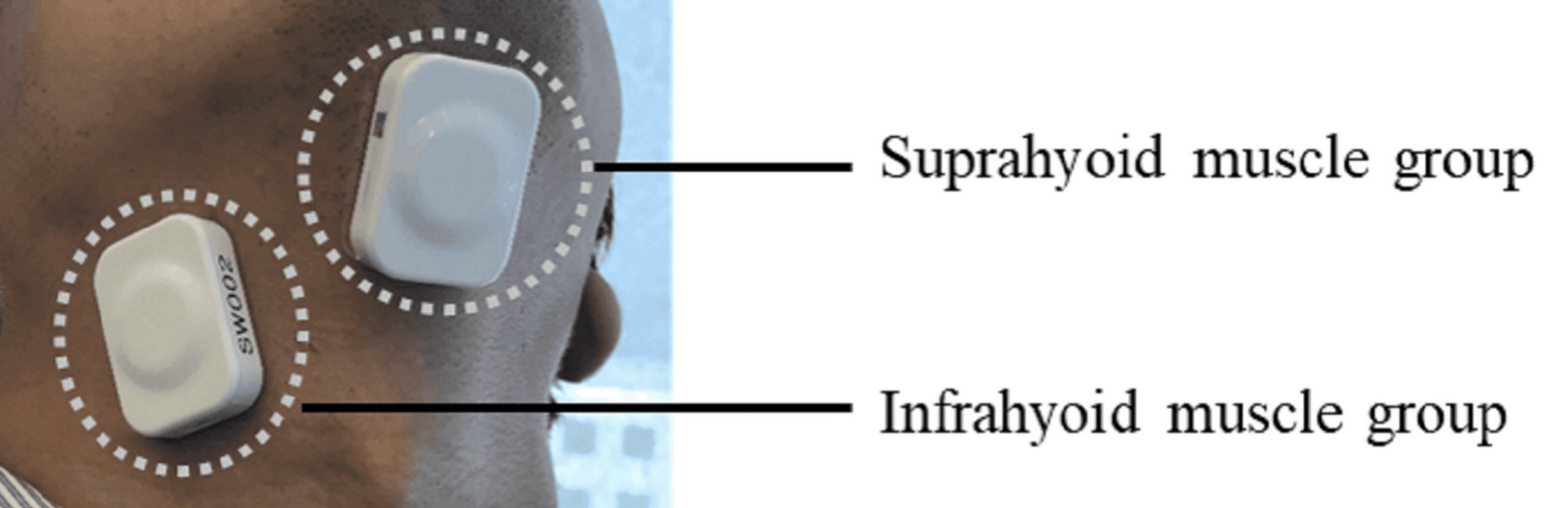
Installation position of surface electromyogram

Surface electromyographic activity was measured using TS-MYO (Trunk Solution Co., Ltd., Tokyo, Japan) at two locations: the suprahyoid muscle group and the infrahyoid muscle group (Figure 1). Electromyographic activity was measured at rest (blank), with water (control), and with each capsule. The resting measurements were performed to visually check whether the measurements were stable and to confirm that the electromyographic measurements were being carried out without any problems. Electromyography was performed three times in total when taking water. Electromyogram measurements were taken at 1,000 points per second, starting five seconds before capsule ingestion and collecting a total of 10 seconds of electromyogram data.

Questionnaire method

**Figure 2:**
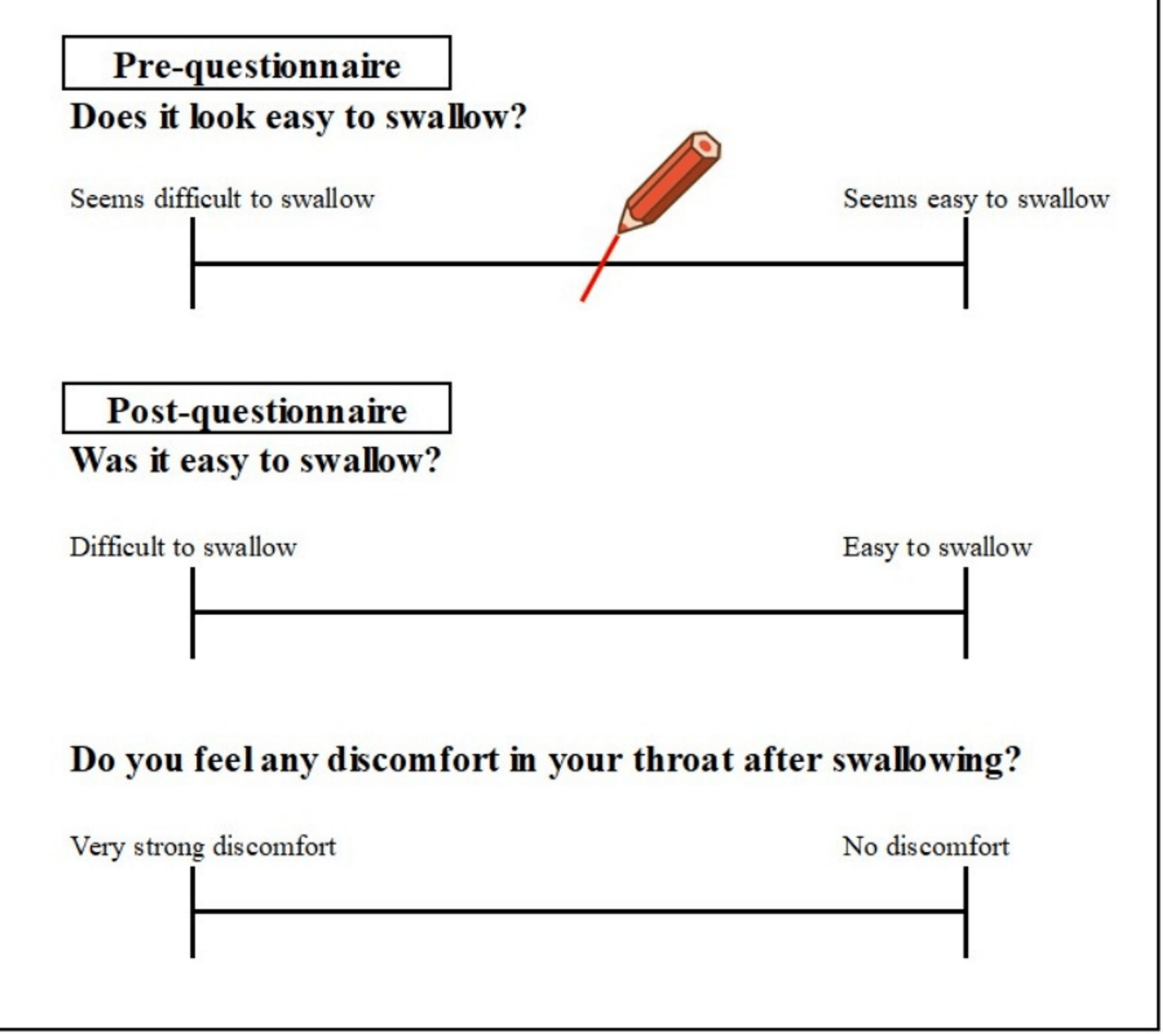
Sample pre- and post-questionnaires in Visual Analogue Scale (VAS) format Image created by the authors of this study.

The questionnaire was carried out using the Visual Analogue Scale (VAS) format with a pre-questionnaire and post-questionnaire (Figure 2) [12, 15, 17]. The pre-questionnaire asked subjects about their impression of the ease of swallowing the medicine. The post-questionnaire investigated the ease of swallowing the medicine and any discomfort in the throat after taking the medicine.

Analysis of the electromyogram

**Figure 3:**
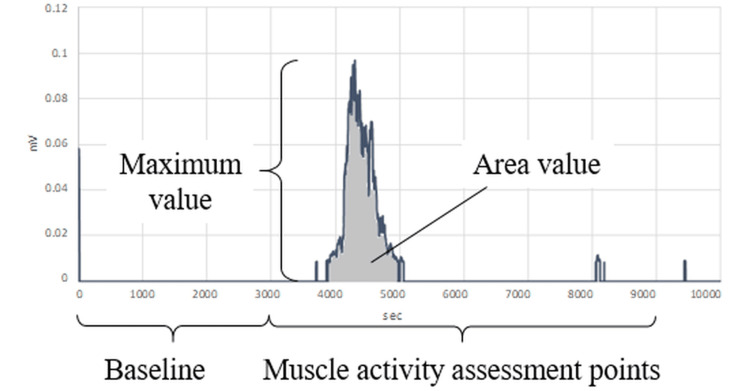
Electromyogram analysis method

For the electromyogram measurement, the frequency range of 50 Hz - 450 Hz was adopted, and the root mean square (RMS) process was carried out. The first three seconds of the data acquired for 10 seconds were used as the baseline, and the mean and standard deviation (S.D.) of the baseline were calculated. Points above the baseline mean +2 S.D. were adopted as muscle activity assessment points, and the integral (area values) and maximum values of muscle activity amounts at the points adopted were calculated (Figure 3).

The average values and S.D. for all subjects were calculated for the area values and maximum values when water and each capsule were taken. The data from three water intake trials were averaged.

Since there was a large variability in the calculated values between subjects, including the data when water was taken, the ratio of the data when each capsule was taken to the data when water was taken for each subject was calculated (the ratio to water), and the average value and S.D. were calculated.

Analysis of questionnaires

In the pre-questionnaire, a score of 0 was assigned to "seems difficult to swallow" and a score of 10 to "seems easy to swallow," and a VAS score was calculated from the intersection of the subjects’ responses. A VAS score was also calculated for the post-questionnaire. VAS scoring of the individual questionnaires was tabulated, and the mean and S.D. of the scores of all subjects were calculated.

Statistical analysis

Electromyographic measurements (area and maximum) were plotted against capsule volume, and each questionnaire result in a scatter plot. The volume and questionnaire score of each capsule were also plotted. In addition, scores by capsule number were plotted for the impression of ease of swallowing medication from the prior questionnaire and for ease of swallowing medication from the posterior questionnaire. Correlation coefficients were calculated (Microsoft Excel 2016, Microsoft Corporation, Redmond, Washington, United States) in all plotting analyses and assessed for the strength of correlation.

The reliability of the electromyography measurement results was evaluated using the intraclass correlation coefficient (ICC), and the ICC and 95% confidence interval were calculated.

## Results

Electromyography results

**Figure 4:**
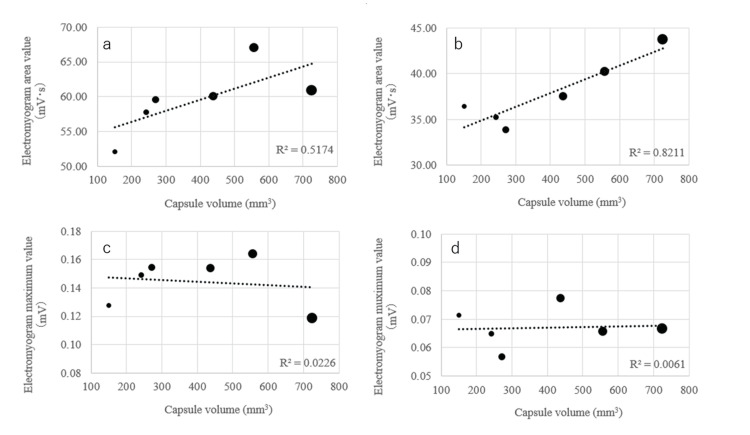
Correlation plots between electromyogram measurement results (measured values) and capsule volume (N=40, average) Correlation plots between capsule volume and actual measured area value of suprahyoid muscles (ICC:0.944 (0.905-0.964)) (a) and infrahyoid muscles (ICC:0.960 (0.935-0.976)) (b) are displayed. Correlation plots between capsule volume and actual measured maximum value of suprahyoid muscles (ICC:0.941 (0.898-0.962)) (c) and infrahyoid muscles(ICC:0.954 (0.923-0.971)) (d) are displayed. For both muscles, a correlation was observed between capsule volume and the electromyogram area value, but there was no correlation between the maximum electromyogram value and capsule volume.

**Figure 5:**
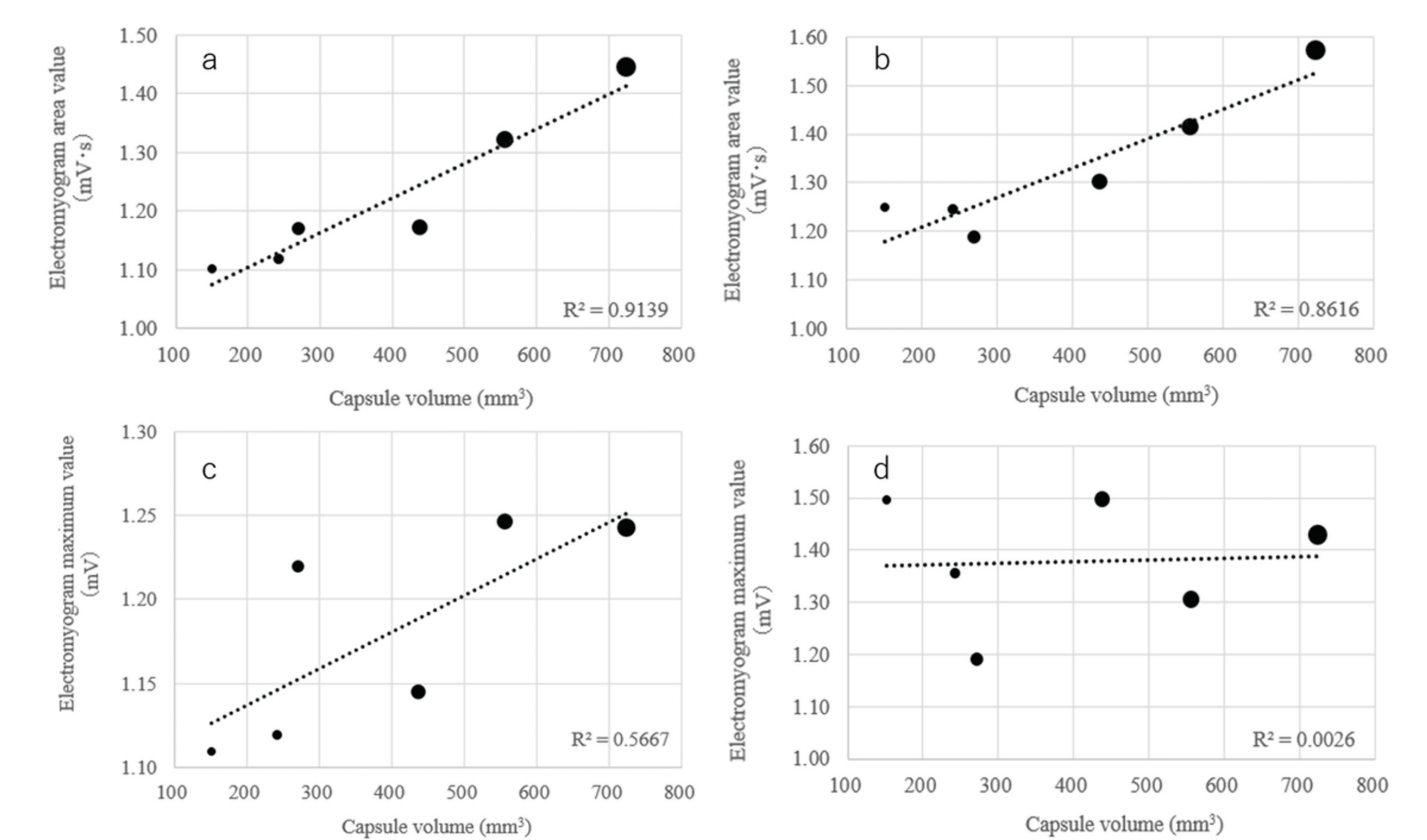
Correlation plots between electromyogram measurement results (ratio to water) and capsule volume (N=40, average) Correlation plots of capsule volume and water ratio of area value for the suprahyoid muscle (ICC:0.831 (0.737-0.902)) (a) and infrahyoid muscle (ICC:0.924 (0.879-0.955)) (b) are displayed. Correlation plots of capsule volume and water ratio of maximum value for the suprahyoid muscle (ICC:0.806 (0.672-0.877)) (c) and infrahyoid muscle (ICC:0.942 (0.901-0.963)) (d) are displayed. For both muscles, electromyogram area values indicated a higher correlation with capsule volume than the maximum electromyogram values.

Table 3 shows the actual measurements and the ratio to water of the suprahyoid and infrahyoid muscle groups of the 40 subjects. There was a relationship between area values of muscle activity and capsule size, but no correlation between maximum values of muscle activity and capsule size (Figure 4). There was a strong correlation between area values and capsule size for the ratio to water, while the correlation between maximum values and capsule size was not high (Figure 5). The reliability of the electromyography measurement results was evaluated using ICC, and the results showed good reliability (>0.75) in all analyses (Figures 4, 5). Correlations were also found between muscle activity and questionnaire results.

**Table 3:** Electromyogram results (N=40, Mean±S.D.)

Measurement type	Muscle group	Parameter	#5	#4	#3	#2	#1	#0
Actual measurement	Suprahyoid muscle group	Area value (mV･s)	52.11	57.77	59.51	60.01	66.93	60.84
			±36.14	±68.65	±56.36	±65.75	±67.57	±39.50
		Maximum value (mV)	0.13	0.15	0.15	0.15	0.16	0.12
			±0.18	±0.35	±0.26	±0.35	±0.34	±0.13
	Infrahyoid muscle group	Area value (mV･s)	36.45	35.27	33.84	37.52	40.21	43.69
			±31.83	±26.03	±23.35	±28.93	±27.39	±36.76
		Maximum value (mV)	0.07	0.06	0.06	0.08	0.07	0.07
			±0.10	±0.07	±0.04	±0.10	±0.06	±0.06
Ratio to water	Suprahyoid muscle group	Area value ratio	1.12	1.11	1.18	1.19	1.34	1.44
			±0.33	±0.37	±0.41	±0.36	±0.59	±0.73
		Maximum value ratio	1.12	1.11	1.23	1.16	1.25	1.25
			±0.41	±0.36	±0.44	±0.37	±0.53	±0.51
	Infrahyoid muscle group	Area value ratio	1.26	1.23	1.19	1.31	1.42	1.58
			±0.93	±0.66	±0.42	±0.84	±0.71	±1.26
		Maximum value ratio	1.50	1.35	1.19	1.50	1.30	1.44
			±2.31	±1.46	±0.40	±1.58	±1.07	±1.39

Questionnaire results

**Figure 6:**
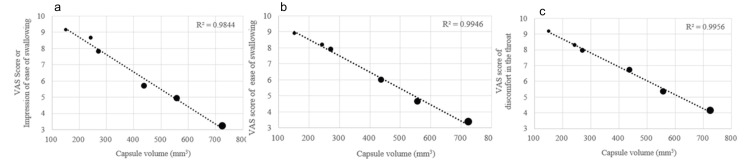
Correlation plots between questionnaire results and capsule volume (N=40, average) Correlation plots between capsule volume and questionnaire results of impression of ease of swallowing (a), actual ease of swallowing (b), and discomfort in the throat after swallowing (c) are displayed. All questionnaire results show a high negative correlation with capsule volume. VAS: Visual Analogue Scale

The VAS scores for each questionnaire for the 40 subjects are shown in Table 4. The questionnaire results for apparent ease of swallowing, actual ease of swallowing, and discomfort in the throat after taking the drug were all strongly correlated with capsule volume (Figure 6).

**Table 4:** Questionnaire results (N=40, Mean±S.D.)

		#5	#4	#3	#2	#1	#0
Pre-questionnaire	Impression of ease of swallowing	9.15	8.67	7.81	5.69	4.91	3.20
		±1.55	±1.38	±1.83	±2.34	±2.47	±2.59
Post-questionnaire	Ease of swallowing	8.93	8.20	7.91	6.00	4.64	3.35
		±1.13	±1.93	±1.77	±2.46	±2.57	±2.22
	Discomfort in the throat	9.19	8.30	7.96	6.72	5.33	4.14
		±0.91	±2.09	±1.91	±2.66	±3.01	±2.83

**Figure 7:**
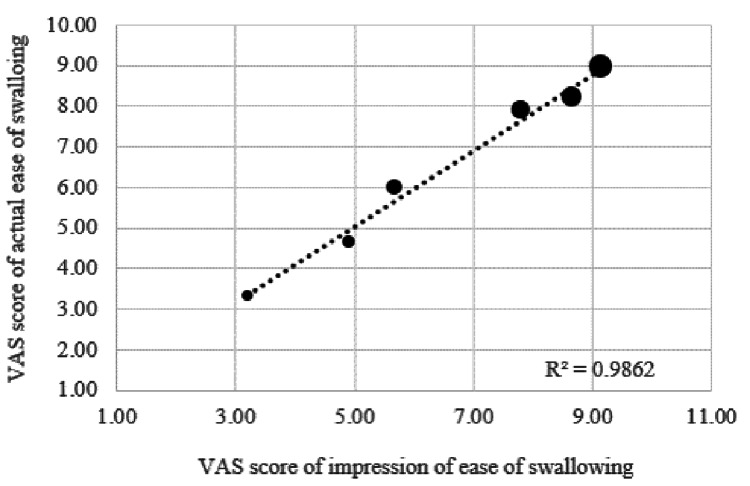
Correlation plot between impression of ease of swallowing and actual ease of swallowing (N=40, average) VAS: Visual Analogue Scale

Apparent ease of swallowing (pre-questionnaire) and actual ease of swallowing (post-questionnaire) were highly correlated (Figure 7).

Correlation between electromyography and questionnaire results

The correlation between the results of electromyography and the results of each questionnaire was also high in the suprahyoid and infrahyoid muscle groups. The ratio to water had a higher correlation coefficient than the measured value, and the area value had a higher correlation coefficient than the maximum value (Table 5).

**Table 5:** Correlation coefficient (R2) between electromyogram analysis results and various questionnaires

Data type	Correlation coefficient		Suprahyoid muscle group		Infrahyoid muscle group	
			Area value	Maximum value	Area value	Maximum value
Actual values	Pre-questionnaire	Impression of ease of swallowing	0.507	0.0157	0.7855	0.0189
	Post-questionnaire	Ease of swallowing	0.5478	0.0131	0.8268	0.0126
		Discomfort in the throat	0.5730	0.0114	0.7999	0.0012
Ratio to water	Pre-questionnaire	Impression of ease of swallowing	0.8656	0.5534	0.8079	0.007
	Post-questionnaire	Ease of swallowing	0.8959	0.5595	0.8537	0.0045
		Discomfort in the throat	0.9219	0.615	0.8346	0.0000

## Discussion

Questionnaire assessment is an important tool in the study of medication swallowability, as shown in a number of reports [7-9]. However, questionnaire assessment generally has problems in ensuring objectivity. It is important to establish methods other than questionnaires to assess the ease of taking medication. Therefore, in this study, we evaluated the usefulness of measuring the muscle activity of swallowing-related muscles as an assessment system for the ease of taking medication.

In this study, it was shown that the amount of muscle activity of swallowing-related muscles increased with the increase in capsule volume and that there was a high correlation between the amount of muscle activity of swallowing-related muscles and the capsule volume. As in previous reports [15, 16], the questionnaire results of this study also suggest that the difficulty of swallowing increases as the capsule volume increases. It has been reported that increased activity in swallowing-related muscles indicates difficulty in swallowing, and this study confirmed a similar trend [11]. In this study, there was a high correlation between the results of the swallowing questionnaire and muscle activity, and there was also a high correlation between muscle activity and capsule volume, leading to the conclusion that muscle activity of swallowing-related muscles is useful for evaluating the difficulty of swallowing medication due to capsule size.

We examined the selection of muscle groups to be evaluated, the evaluation parameters, and the evaluation methods. Although the suprahyoid muscle group and the infrahyoid muscle group were selected for evaluation according to previous literature [12], the results of this study also showed that both muscle groups can be adopted as evaluation targets. For the evaluation parameters, maximum value, total amount, muscle activity period, etc., were evaluated in previous research [11, 12], and for this study, it was determined that the total amount (area) of muscle activity was suitable. In cases where the difference in swallowability is slight, such as due to a difference in capsule volume being small, we considered that the maximum value had low detection sensitivity, taking into account the variability of the data. Therefore, it is considered that evaluation using the total amount, including the muscle activity period, is suitable. In addition, it was proven that evaluation with high sensitivity was possible by calculating the ratio to the amount of muscle activity in ingesting water set as a baseline. We believe that this is due to the large variability in the amount of muscle activity during swallowing, which differs among individuals.

This study used a questionnaire to assess both the apparent ease of swallowing and the actual ease of swallowing for capsule sizes 0-5. As previously reported [15], the apparent ease of swallowing was shown to increase and decrease according to capsule size, but it was also similar when it was actually taken, indicating a high correlation. In assessing the ease of swallowing attributable to size, it was not necessary to take the drug in practice, and by assessing the apparent ease of swallowing, it was judged that the ease of swallowing could be sufficiently evaluated.

Improving medication adherence in treatment is an important global challenge. Developing easy-to-swallow medicines is essential in improving medication adherence, and establishing methods to evaluate easy-to-swallow medicines with high precision is also important in developing such easy-to-swallow medicines. We believe that the evaluation of the surface muscle activity of swallowing-related muscles carried out in this study is a useful method for assessing the ease of swallowing a drug in the development of a drug that is easy to swallow, because it is a less invasive and convenient method for subjects. However, in the evaluation system constructed in this study, it was only proven that swallowability can be evaluated mainly based on the factor of size. Humans combine many factors to assess the ease of swallowing drugs. It has been reported that the surface muscle activity evaluation system for swallowing-related muscles can evaluate swallowing disorders caused by factors such as stiffness and posture [11-13], and together with the results of this study, we believe that this system can evaluate the ease of swallowing caused by many factors. However, whether this evaluation system can evaluate a dosage form other than that of capsules, whether multiple factors such as size and taste can be evaluated comprehensively, etc., should be examined in the future. Since this study was conducted on healthy adults aged 20-39 years, whether it is feasible also in children for reasons such as minimization of activities other than swallowing as much as possible, or whether it is adaptable to elderly or dysphagic subjects who are considered to have low muscle activity, should be further investigated. It is expected that the usefulness and limitations of this evaluation system will be judged by future research and that it will become a suitable alternative method as an evaluation system for the swallowability of drugs in medical treatment. It is important to establish a method for appropriately evaluating the ease of administration, including the usage of this evaluation system in combination with questionnaire evaluation.

## Conclusions

Numerous studies have shown that reduced medication adherence leads to a lowering of the treatment effect, and the improvement in adherence to the treatment is a very important problem worldwide. Medication non-adherence arises from multiple factors, among which difficulty in swallowing has been identified as a contributing cause. Therefore, while developing a medication, it is crucial to try to make it easy to swallow. It is also crucial to accurately assess how easy it is to swallow a medication.

The authors attempted to develop a system to evaluate the swallowability of medications by measuring the electromyographic activity of swallowing-related muscles. The results of this study, in which healthy adults were given capsules, showed a correlation between the area value of muscle activity and capsule size. Furthermore, a correlation was also shown between the results of a questionnaire about ease of swallowing and the amount of muscle activity (area value).

These results indicate that evaluating the area value of superficial muscle activity of the suprahyoid and infrahyoid muscles, especially using the ratio to muscle activity during drinking water, is a useful method for evaluating the swallowability of medications.
